# Urinary creatinine estimation as an important analytical limitation of semi-quantitative albumin-to-creatinine ratio strip testing: a diagnostic accuracy study

**DOI:** 10.3389/fmedt.2026.1913468

**Published:** 2026-10-29

**Authors:** Piyawan Bunpo, Pharisa Nanthawong, Chananon Chomphusri, Waratchaya Ratchawat, Suwatsin Kittikunnathum, Cheewaporn Tanintheerakul, Rungnapa Peerakam

**Affiliations:** 1 Division of Clinical Chemistry, Department of Medical Technology, Faculty of Associated Medical Sciences, Chiang Mai University, Chiang Mai, Thailand; 2 Clinical Chemistry Laboratory, Diagnostic Service Unit, Faculty of Medicine, Maharaj Nakorn Chiang Mai Hospital, Chiang Mai University, Chiang Mai, Thailand

**Keywords:** albuminuria, diagnostic accuracy studies, immunoturbidimetric assay, point-of-care testing, urinary albumin-to-creatinine ratio

## Abstract

**Background:**

Albuminuria is a key biomarker for chronic kidney disease and cardiovascular risk. Point-of-care urine albumin-to-creatinine ratio (UACR) testing may improve access to chronic kidney disease screening. However, limited evidence is available regarding the agreement of the individual albumin and creatinine components of semi-quantitative UACR strip tests with quantitative laboratory measurements.

**Methods:**

We evaluated 400 spot urine specimens using the Dirui H13-Cr semi-quantitative UACR strip and compared results with quantitative immunoturbidimetric UACR measurement performed on a Roche Cobas c503 analyzer. Agreement was assessed using Cohen's kappa coefficients, including category-specific agreement according to the KDIGO A1, A2, A3 albuminuria categories, and diagnostic performance was evaluated using sensitivity, specificity, predictive values, likelihood ratios, and diagnostic odds ratio (DOR).

**Results:**

Agreement between strip-based and quantitative measurements was substantial for urinary albumin (*κ*=0.691; 95% CI, 0.632–0.751) but considerably lower for urinary creatinine (*κ*=0.377; 95% CI, 0.331–0.423). Despite this disparity, UACR classification demonstrated substantial agreement (*κ*=0.627; 95% CI, 0.541–0.714). The findings suggest that semi-quantitative creatinine estimation is the primary analytical determinant of UACR misclassification. For detection of abnormal albuminuria (UACR ≥30 mg/g), the strip achieved a sensitivity of 83.66% and specificity of 83.22%. The PPV was 89.96% and NPV was 73.91%. Category-specific analysis showed that 131 of 177 A2 specimens (74.0%) were classified as A2 by the strip, whereas 64 of 80 A3 specimens (80.0%) were classified as A2 rather than A3.

**Conclusion:**

The Dirui H13-Cr strip demonstrated acceptable performance for albuminuria screening. However, semi-quantitative urinary creatinine estimation represented an important analytical limitation affecting UACR classification accuracy. Future development of strip-based UACR technologies should prioritize improved creatinine measurement to enhance diagnostic performance.

## 1 Introduction

Microalbuminuria represents an early and sensitive marker of glomerular injury and is a well-established predictor of progressive renal disease and adverse cardiovascular outcomes, particularly in individuals with diabetes mellitus and hypertension. Even low-grade increases in urinary albumin excretion are associated with endothelial dysfunction and increased cardiovascular and renal risk, underscoring the clinical importance of early detection and intervention (1–4). Current KDIGO recommendations classify albuminuria into three categories according to the urine albumin-to-creatinine ratio (UACR): A1 (<30 mg/g), A2 (30–299 mg/g), and A3 (≥300 mg/g). These categories provide clinically relevant information for identifying individuals at increased risk of adverse kidney and cardiovascular outcomes (5).

Quantitative laboratory measurement of urinary albumin and creatinine provides the basis for UACR assessment in routine clinical practice. Immunoturbidimetric and nephelometric methods are widely used for urinary albumin measurement, while enzymatic or other standardized methods are available for urinary creatinine measurement. These laboratory methods provide quantitative results with established analytical performance and are commonly used for diagnosis, monitoring, and confirmation of albuminuria (5, 6). As a result, immunoassay-based measurements of urinary albumin or UACR are frequently employed as the diagnostic reference standard in studies evaluating the performance of screening tests for microalbuminuria (7–11).

To overcome limitations related to cost, infrastructure, and turnaround time associated with centralized laboratory testing, point-of-care microalbumin strips and semi-quantitative UACR cassette tests have been developed (8, 9, 12–15). These tests offer rapid, near-patient results, require minimal training and equipment, and allow same-visit clinical decision-making in primary care settings (16). Several commercially available point-of-care systems, including Micral-Test II, Clinitek Microalbumin, HemoCue urine albumin, and semi-quantitative UACR cassettes, have demonstrated moderate to high sensitivity in various clinical cohorts. Reported sensitivities generally range from approximately 79% to over 95%, with specificities ranging more widely depending on device type, cutoff values, and study population (16–19).

However, published head-to-head comparisons between microalbumin strips and laboratory immunoassays have yielded heterogeneous results. While some studies reported excellent sensitivity and negative predictive value, supporting the utility of strip-based testing as a screening tool, others demonstrated reduced specificity and lower diagnostic discrimination compared with quantitative UACR or albumin measurements obtained by immunoassay methods (16, 17). Systematic reviews and meta-analyses have further highlighted substantial variability in diagnostic accuracy among different dipstick brands and testing formats, indicating an absence of consensus regarding the reliability of semi-quantitative strip testing for routine microalbuminuria screening (16, 20).

Several limitations in the existing literature contribute to this uncertainty, including heterogeneity in reference standards (24-hour urinary albumin excretion, albumin concentration, or UACR), variability in diagnostic thresholds, population-specific prevalence effects, and pre-analytical factors such as sample handling and interfering substances (6, 16). Despite the operational advantages of point-of-care testing, direct comparisons using standardized cutoffs and contemporary immunoassay reference methods in representative clinical populations remain limited (18, 20, 21).

Although most evaluations of strip-based UACR testing focus on overall diagnostic accuracy, relatively few studies have examined which analytical component contributes most to classification error. Because UACR is calculated from both urinary albumin and creatinine concentrations, inaccuracies in either measurement may affect clinical interpretation. Understanding whether albumin or creatinine estimation constitutes the dominant source of error is important for future development of point-of-care UACR technologies and may explain differences in performance observed among commercially available devices. Among commercially available semi-quantitative UACR strip systems, the Dirui H13-Cr provides simultaneous semi-quantitative estimation of urinary albumin and creatinine and reports the albumin-to-creatinine ratio based on these measurements. The availability of the Dirui H13-Cr through a local distributor also made the system readily accessible for evaluation under routine laboratory conditions. Therefore, this study aimed not only to evaluate the diagnostic performance of the Dirui H13-Cr semi-quantitative UACR strip but also to explore which analytical component may contribute to UACR misclassification by separately examining agreement for urinary albumin and creatinine measurements.

## 2 Materials and methods

### 2.1 Study design and sample selection

This cross-sectional diagnostic accuracy study was conducted at the Clinical Chemistry Laboratory, Diagnostic Service Unit, Maharaj Nakorn Chiang Mai Hospital, Faculty of Medicine, Chiang Mai University, and the Division of Clinical Chemistry, Department of Medical Technology, Faculty of Associated Medical Sciences, Chiang Mai University. This study was conducted and reported according to the Standards for Reporting of Diagnostic Accuracy Studies (STARD 2015). Spot midstream urine samples were collected from individuals undergoing routine health check-ups between November 2025 and February 2026. Each urine specimen represented a separate participant. All samples were anonymized prior to analysis, and no personal identifiers were recorded.

### 2.2 Sample size calculation and classification

Sample size was calculated to estimate the sensitivity and specificity of the microalbumin strip test with a 95% confidence level (Z = 1.96). Expected sensitivity and specificity were assumed to be 85% and 90%, respectively, with an acceptable margin of error of ±5%. Based on these assumptions, a minimum of 139 negative samples (normoalbuminuria) was required to estimate specificity and at least 196 positive samples (microalbuminuria and clinical albuminuria combined) to estimate sensitivity, resulting in a minimum total sample size of 335 specimens.

UACR categories were defined according to the quantitative reference method using current KDIGO terminology: A1, UACR <30 mg/g; A2, UACR 30–299 mg/g; and A3, UACR ≥300 mg/g (5). The study sample was designed to provide adequate representation across the clinically relevant UACR categories. Consequently, the distribution of UACR categories in the study sample should not be interpreted as the prevalence of albuminuria in a general health-screening population. A total of 400 samples were included, comprising 143 normoalbuminuria, 177 microalbuminuria, and 80 clinical albuminuria samples, after excluding specimens positive for hemoglobin or myoglobin (≥5 mg/dL) or with urine pH ≥9.0.

### 2.3 Ethical considerations

The study was conducted in accordance with the Declaration of Helsinki and was approved by the Ethics Research Committee of the Faculty of Medicine, Chiang Mai University (Study code: None−2568–0763). Because the study used de-identified and anonymized clinical specimens with minimal risk to participants, the requirement for individual informed consent was waived.

### 2.4 Laboratory measurements

#### 2.4.1 Sample preparation and UACR calculation

Urine samples were centrifuged at 3,000 revolutions per minute (kokusan H−36, Japan) for 10 min prior to analysis. The clarified urine was subsequently analyzed using both semi-quantitative and quantitative methods on the same day. Centrifugation was applied uniformly to standardize sample preparation for comparison of the two analytical methods. Because centrifugation may not be routinely performed before decentralized point-of-care urine testing, this sample-preparation procedure was considered when interpreting the generalizability of the findings to routine point-of-care practice.

#### 2.4.2 Semi-quantitative microalbumin strip test

The microalbumin strip test (Dirui H13-Cr; Dirui Industrial Co., Ltd., China) was performed according to the manufacturer's instructions. Creatinine measurement was based on a colorimetric reaction with 3,5-dinitrobenzoic acid under alkaline conditions, corresponding to concentrations of 50, 100, 200, and 300 mg/dL. Albumin was detected using a sulfonephthalein dye reaction, producing semi-quantitative results across five concentration ranges (10, 30, 80, and 150 mg/L).

Strip reactions were interpreted visually according to the manufacturer's instructions. The corresponding semi-quantitative albumin and creatinine values were used to calculate the strip-derived UACR. The calculated UACR was subsequently classified using the same clinical thresholds as the reference method: A1 (<30 mg/g), A2 (30–299 mg/g), and A3 (≥300 mg/g). Operators performing the strip testing were blinded to the quantitative laboratory results.

#### 2.4.3 Quantitative reference assays

Quantitative assays were performed using the Roche Cobas® c503 clinical chemistry analyzer (Roche Diagnostics, Mannheim, Germany). Urinary albumin concentration was measured by an immunoturbidimetric method based on antigen-antibody complex formation (Tina-quant® Albumin Gen.2, Roche Diagnostics), with turbidity measured photometrically and directly proportional to albumin concentration. Urinary creatinine was determined using an enzymatic assay (Creatinine Plus ver.2, Roche Diagnostics) involving sequential hydrolysis by creatininase and creatinase, followed by sarcosine oxidation to generate a chromogenic compound. Absorbance was measured bichromatically at 700/546 nm. Calibration and internal quality control were conducted using manufacturer-provided reagents and control materials. For the quantitative method, UACR was calculated as:

UACR (mg/g) = Urine albumin (mg/L) × 1,000 ÷ Urine creatinine (g/L)

#### 2.4.4 Diagnostic accuracy analysis

Sensitivity, specificity, positive predictive value, and negative predictive value were calculated using standard formulas (22). Quantitative UACR measurement served as the reference method for all diagnostic accuracy analyses.

### 2.5 Statistical analysis

Agreement between semi-quantitative strip testing and quantitative measurements for urinary albumin, urinary creatinine, and UACR, was evaluated using cross-tabulation and quadratic weighted Cohen's kappa coefficients with 95% confidence intervals (CIs), accounting for the ordinal nature of concentration categories. For dichotomous UACR classification (<30 vs. ≥ 30 mg/g), unweighted Cohen's kappa was used because the outcome consisted of two categories without an ordinal weighting structure.

Diagnostic performance of the semi-quantitative UACR strip was assessed using the quantitative immunoassay as the reference standard. Sensitivity, specificity, positive predictive value (PPV), and negative predictive value (*N*PV) were calculated with 95% CIs. A two-sided *p* value <0.05 was considered statistically significant. All analyses were conducted using SPSS version 17.0 statistical software.

## 3 Results

### 3.1 Agreement between semi-quantitative and quantitative measurements

A total of 400 urine specimens were analyzed using the Dirui H13-Cr semi-quantitative strip and compared with quantitative laboratory measurements. For urinary albumin classification, agreement between the semi-quantitative strip and quantitative immunoturbidimetric assay was substantial (weighted *κ*=0.691, 95% CI, 0.632–0.751; *p* < 0.001) (Table 1). Exact agreement was observed in 254 of 400 samples (63.5%), with most discrepancies occurring between adjacent albumin categories.

**Table 1:** Agreement between semi-quantitative strip test and quantitative immunoassay for urine albumin concentration categories (*N* = 400).

		Quantitative albumin (mg/L)			Total
		<30	30–149	≥150	
**Semi-quantitative albumin (mg/L)**	<30	70	21	2	93
	30–149	48	78	50	176
	≥150	0	25	106	131
**Total**		118	124	158	400

Data are presented as number of samples. Agreement was assessed using quadratic weighted Cohen's kappa to account for the ordered nature of urine albumin concentration categories. The quantitative immunoassay served as the reference method. Weighted kappa=0.691 (95% CI, 0.632–0.751; *p* < 0.001).

Agreement for urinary creatinine classification was lower (weighted *κ*=0.377, 95% CI, 0.331–0.423; *p* < 0.001) (Table 2). Misclassification was observed across multiple creatinine categories, indicating reduced concordance between semi-quantitative and quantitative creatinine measurements.

**Table 2:** Agreement between semi-quantitative strip test and quantitative method for urine creatinine concentration categories (*N* = 400).

		Quantitative creatinine (mg/dL)					Total
		<50	50–99	100–199	200–299	≥300	
**Semi-quantitative creatinine (mg/dL)**	<50	15	3	0	0	0	18
	50–99	56	43	4	0	1	104
	100–199	19	24	6	0	0	49
	200–299	10	35	14	0	0	59
	≥300	4	37	88	35	6	170
**Total**		104	142	112	35	7	400

Data are presented as number of samples. Agreement between semi-quantitative strip testing and quantitative creatinine measurement was evaluated using quadratic weighted Cohen's kappa. The quantitative method was used as the reference standard. Weighted kappa=0.377 (95% CI, 0.331–0.423; *p* < 0.001).

For three-category UACR classification, substantial agreement was observed between the semi-quantitative strip and quantitative reference method (weighted *κ*=0.627, 95% CI, 0.541–0.714; *p* < 0.001) (Table 3). Exact agreement was achieved in 266 of 400 samples (66.5%). Most discordant results occurred between adjacent UACR categories, while major discrepancies were uncommon.

**Table 3:** Agreement between semi-quantitative strip test and quantitative immunoassay for urine albumin-to-creatinine ratio (UACR) categories (*N* = 400).

		Quantitative UACR **(**mg/g**)**			Total
		A1 (<30)	A2 (30–299)	A3 (≥300)	
**Semi-quantitative UACR (mg/g)**	A1 (<30)	119	42	0	161
	A2 (30–299)	24	131	64	219
	A3 (≥300)	0	4	16	20
**Total**		143	177	80	400

Data are presented as number of samples. Agreement for urine albumin-to-creatinine ratio categories was assessed using quadratic weighted Cohen's kappa. UACR classification was based on the quantitative immunoassay reference method. Weighted kappa=0.627 (95% CI, 0.541–0.714; *p* < 0.001).

### 3.2 Category-specific UACR classification

The cross-tabulation of UACR categories demonstrated that 119 of 143 A1 specimens were correctly classified as A1 by the strip. Among the 177 reference-defined A2 specimens, 131 (74.0%) were classified as A2 and 4 as A3 by the strip, whereas 42 were classified as A1. Thus, 135 of 177 A2 specimens (76.3%) were identified as abnormal by the strip (Table 3).

Among the 80 reference-defined A3 specimens, 16 were correctly classified as A3, whereas 64 were classified as A2. Therefore, the strip correctly identified 20.0% of A3 specimens, while 80.0% were classified one category lower as A2.

These findings indicate that the strip was generally capable of identifying abnormal albuminuria but had more limited ability to distinguish the severity of albuminuria, particularly between A2 and A3.

### 3.3 Diagnostic performance for detection of abnormal albuminuria

Using a UACR threshold of ≥30 mg/g as the reference definition of abnormal albuminuria, 257 samples were classified as positive and 143 as negative by the quantitative method (Table 4). The semi-quantitative strip correctly identified 215 true-positive and 119 true-negative samples, while 42 false-negative and 24 false-positive results were observed. Overall agreement between methods was substantial (unweighted *κ*=0.651, 95% CI, 0.587–0.714; *p* < 0.001).

**Table 4:** Diagnostic agreement between semi-quantitative strip test and quantitative immunoassay for detection of abnormal albuminuria (UACR ≥30 mg/g) (*N* = 400).

		Quantitative UACR		Total
		A1 (<30 mg/g)	A2/A3 (≥30 mg/g)	
**Semi-quantitative UACR (mg/g)**	Strip Negative (<30 mg/g)	119 (TN)	42 (FN)	161
	Strip Positive (≥30 mg/g)	24 (FP)	215 (TP)	239
**Total**		143	257	400

Data are presented as number of samples. TP, true positive; TN, true negative; FP, false positive; FN, false negative. Diagnostic agreement was evaluated using a dichotomous UACR classification (<30 vs. ≥ 30 mg/g). Unweighted Cohen's kappa was used to assess overall agreement between methods. Cohen's kappa=0.651(95% CI, 0.587–0.714; *p* < 0.001).

Diagnostic performance metrics are summarized in Table 5. The strip demonstrated a sensitivity of 83.66% (95% CI, 78.53–87.94) and specificity of 83.22% (95% CI, 76.02–88.94). The positive predictive value was 89.96% (95% CI, 85.41–93.44), whereas the negative predictive value was 73.91% (95% CI, 66.44–80.45). Overall accuracy was 83.50%. The positive likelihood ratio was 4.99, the negative likelihood ratio was 0.20, and the diagnostic odds ratio was 25.4.

**Table 5:** Diagnostic performance of the Dirui H13-Cr semi-quantitative strip for detection of abnormal albuminuria using quantitative UACR as the reference standard (*N* = 400).

Parameter	Dirui H13-Cr
Sensitivity (%)	83.66 (95% CI, 78.53–87.94)
Specificity (%)	83.22 (95% CI, 76.02–88.94)
Positive predictive value (%)	89.96 (95% CI, 85.41–93.44)
Negative predictive value (%)	73.91 (95% CI, 66.44–80.45)
Overall accuracy (%)	83.50 (95% CI, 79.53–86.96)
Positive likelihood ratio (LR+)	4.99 (95% CI, 3.44–7.24)
Negative likelihood ratio (LR−)	0.20 (95% CI, 0.15–0.26)
Diagnostic odds ratio (DOR)	25.4 (95% CI, 15.4–41.8)

Diagnostic performance metrics were calculated using the quantitative immunoturbidimetric UACR assay as the reference standard. Positive likelihood ratio (LR+) was calculated as sensitivity/(1-specificity), negative likelihood ratio (LR−) as (1-sensitivity)/specificity, and diagnostic odds ratio (DOR) as LR+/LR−. Overall accuracy was calculated as (TP + TN)/N.

## 4 Discussion

### 4.1 Overall diagnostic performance of the semi-quantitative UACR strip

This study evaluated the diagnostic performance of the Dirui H13-Cr semi-quantitative UACR strip for detecting albuminuria using a quantitative immunoturbidimetric assay as the reference method. The strip demonstrated good overall diagnostic performance, with a sensitivity of 83.66%, specificity of 83.22%, positive predictive value of 89.96%, and negative predictive value of 73.91%. Furthermore, substantial agreement was observed for albumin classification and UACR classification, supporting the potential role of the strip as an initial screening tool for albuminuria.

Albuminuria is a well-established marker of kidney damage and an independent predictor of chronic kidney disease (CKD) progression, cardiovascular events, and mortality. Current KDIGO guidelines recommend assessment of albuminuria for CKD detection, staging, and risk stratification, highlighting the importance of accessible screening approaches, particularly in populations at increased risk of CKD. The increasing prevalence of diabetes, hypertension, and aging populations worldwide further emphasizes the need for practical methods that facilitate early identification of individuals requiring further nephrological evaluation and intervention (5, 23).

The diagnostic performance observed in our study is consistent with previous investigations evaluating semi-quantitative UACR testing. Nah et al. reported sensitivities ranging from 80% to 92% and specificities ranging from 91.6% to 94% when comparing a UACR strip test with quantitative laboratory measurement in individuals with prediabetes and diabetes (24). Similarly, McTaggart et al. demonstrated that point-of-care UACR testing could effectively identify albuminuria in primary care settings while reducing barriers associated with centralized laboratory testing (20). More recently, Currin et al. reported acceptable diagnostic accuracy of semi-quantitative UACR testing in a resource-limited primary care setting, supporting the use of point-of-care approaches where laboratory infrastructure is limited (25). Collectively, these findings suggest that semi-quantitative UACR strips can provide clinically meaningful screening information across diverse healthcare environments.

### 4.2 Urinary creatinine estimation as an important analytical limitation

The most important finding of this study was the marked discrepancy between albumin and creatinine agreement. While urinary albumin classification demonstrated substantial agreement with quantitative laboratory measurement (*κ*=0.691), urinary creatinine classification achieved only fair agreement (*κ*=0.377). This finding suggests that creatinine estimation may represent an important analytical limitation of semi-quantitative UACR testing. However, the difference between the two kappa coefficients should be interpreted cautiously. The coefficients were calculated from different numbers and distributions of concentration categories, and therefore cannot be used alone to establish that creatinine is responsible for a specific proportion of UACR misclassification.

This observation has important implications because UACR is a ratio measurement. Even relatively small errors in creatinine categorization may lead to substantial changes in calculated UACR values and subsequent albuminuria classification. Consequently, inaccuracies in creatinine estimation may exert a disproportionately greater influence on clinical interpretation than comparable errors in albumin measurement.

Previous studies have generally reported overall diagnostic accuracy of point-of-care UACR testing without specifically examining the relative contributions of albumin and creatinine measurements to classification performance (9, 12, 19, 26). Our findings suggest that urinary creatinine estimation may be an important area for further improvement in point-of-care UACR technologies. Enhancing the accuracy of creatinine estimation, alongside continued optimization of albumin detection, may help improve the overall agreement and diagnostic performance of semi-quantitative UACR testing.

These findings may also explain why different strip-based UACR platforms often demonstrate variable performance despite using similar albumin detection chemistries. Variability in creatinine estimation may represent an under-recognized source of inter-device differences and warrants further investigation. Importantly, the present study does not establish that creatinine estimation is the sole or definitive cause of UACR misclassification. Rather, the lower agreement observed for creatinine identifies it as a potentially important area for analytical improvement.

### 4.3 Category-specific misclassification and clinical implications

Misclassification analysis provides additional insight into the clinical utility of the assay. Among 400 samples, 42 were classified as negative by the strip but positive by the quantitative method, whereas 24 were classified as positive by the strip but negative by the reference assay. Importantly, most discordant results occurred between adjacent albuminuria categories, indicating that disagreement was largely attributable to samples with concentrations near clinical decision thresholds rather than major analytical errors. Similar patterns have been observed in other semi-quantitative albuminuria studies and reflect the inherent limitations of categorical testing around cutoff values (26, 27).

Nevertheless, false-negative results remain clinically relevant because missed albuminuria may delay recognition of CKD and cardiovascular risk. Consequently, negative strip results should be interpreted cautiously in individuals with a high pre-test probability of kidney disease, including patients with diabetes mellitus, hypertension, or established CKD. In contrast, the relatively low number of false-positive results supports the use of positive strip findings as a trigger for confirmatory laboratory-based UACR testing.

The category-specific results provide additional information beyond overall sensitivity and specificity. The strip identified 135 of 177 reference-defined A2 specimens as abnormal, corresponding to 76.3%. This indicates that approximately three-quarters of A2 specimens were recognized as having abnormal albuminuria. However, the strip correctly classified only 16 of 80 reference-defined A3 specimens as A3. The remaining 64 specimens (80.0%) were classified as A2. This downward classification is clinically important because A3 albuminuria represents a substantially higher degree of albuminuria and is associated with greater kidney and cardiovascular risk than A2 albuminuria. Therefore, the ability of a semi-quantitative strip to distinguish albuminuria severity should not be inferred solely from its ability to distinguish normal from abnormal UACR.

The positive predictive value of approximately 90% observed in this study was higher than that reported in several previous investigations (24, 28). This finding is likely attributable to the relatively high prevalence of albuminuria within our study population. As the sample was designed to ensure adequate representation across clinically relevant UACR categories, its UACR distribution should not be interpreted as reflecting the prevalence of albuminuria in a general screening population. Predictive values are strongly influenced by disease prevalence, and therefore similar positive predictive values may not be observed in lower-risk screening populations. Conversely, the negative predictive value observed in our study was lower than those reported in studies involving populations with lower albuminuria prevalence (24, 28–31). These observations underscore the importance of considering disease prevalence and patient selection when interpreting screening performance.

### 4.4 Implications for laboratory medicine and decentralized testing

From a laboratory implementation perspective, our findings support a two-step testing strategy. Semi-quantitative UACR strips offer several practical advantages, including rapid turnaround time, minimal instrumentation, ease of operation, and applicability in decentralized healthcare settings. Positive screening results can subsequently be confirmed using quantitative laboratory-based UACR measurement, thereby combining the accessibility of point-of-care testing with the analytical robustness of standardized laboratory methods. Similar testing algorithms have been proposed for CKD screening programs and may improve access to albuminuria assessment in resource-limited environments (12, 25, 32).

### 4.5 Strengths and limitations

The present study has several strengths. First, it included a relatively large number of specimens representing a broad spectrum of albuminuria severity. Second, the reference method was a standardized quantitative immunoturbidimetric assay routinely used in clinical laboratories. Third, multiple measures of diagnostic accuracy and agreement were evaluated, providing a comprehensive assessment of test performance. However, several limitations should be acknowledged. An important limitation of this study is that only one commercially available semi-quantitative UACR strip system, the Dirui H13-Cr, was evaluated. Therefore, the findings may not be directly generalizable to other semi-quantitative UACR strip products, which may use different analytical principles, reagent formulations, calibration procedures, or approaches to albumin and creatinine estimation. In addition, no direct comparison with other commercially available semi-quantitative UACR strips was performed. The high prevalence of albuminuria may have influenced predictive values. Potential analytical interferences, observer variability, and lot-to-lot variation were not evaluated. Furthermore, the study assessed diagnostic accuracy rather than patient outcomes; therefore, the impact of strip-based screening on CKD progression, cardiovascular events, or healthcare utilization remains uncertain.

## 5 Conclusions

The Dirui H13-Cr semi-quantitative strip demonstrated acceptable performance for albuminuria screening. Beyond overall diagnostic accuracy, this study identified semi-quantitative urinary creatinine estimation as an important analytical limitation affecting UACR classification. These findings suggest that future development of strip-based UACR technologies should prioritize improvements in creatinine measurement to enhance clinical performance and reduce classification errors. In addition, the substantial proportion of A3 specimens classified as A2 indicates that the strip has limited ability to determine albuminuria severity.

## Data Availability

The original contributions presented in the study are included in the article, further inquiries can be directed to the corresponding author.
